# Aortography Keypoint Tracking for Transcatheter Aortic Valve Implantation Based on Multi-Task Learning

**DOI:** 10.3389/fcvm.2021.697737

**Published:** 2021-07-19

**Authors:** Viacheslav V. Danilov, Kirill Yu. Klyshnikov, Olga M. Gerget, Igor P. Skirnevsky, Anton G. Kutikhin, Aleksandr A. Shilov, Vladimir I. Ganyukov, Evgeny A. Ovcharenko

**Affiliations:** ^1^Research Laboratory for Processing and Analysis of Big Data, Tomsk Polytechnic University, Tomsk, Russia; ^2^Department of Experimental Medicine, Research Institute for Complex Issues of Cardiovascular Diseases, Kemerovo, Russia

**Keywords:** keypoint tracking, multi-task learning, transcatheter aortic valve replacement, deep learning—CNN, medical image analysis, aortography

## Abstract

Currently, transcatheter aortic valve implantation (TAVI) represents the most efficient treatment option for patients with aortic stenosis, yet its clinical outcomes largely depend on the accuracy of valve positioning that is frequently complicated when routine imaging modalities are applied. Therefore, existing limitations of perioperative imaging underscore the need for the development of novel visual assistance systems enabling accurate procedures. In this paper, we propose an original multi-task learning-based algorithm for tracking the location of anatomical landmarks and labeling critical keypoints on both aortic valve and delivery system during TAVI. In order to optimize the speed and precision of labeling, we designed nine neural networks and then tested them to predict 11 keypoints of interest. These models were based on a variety of neural network architectures, namely MobileNet V2, ResNet V2, Inception V3, Inception ResNet V2 and EfficientNet B5. During training and validation, ResNet V2 and MobileNet V2 architectures showed the best prediction accuracy/time ratio, predicting keypoint labels and coordinates with 97/96% accuracy and 4.7/5.6% mean absolute error, respectively. Our study provides evidence that neural networks with these architectures are capable to perform real-time predictions of aortic valve and delivery system location, thereby contributing to the proper valve positioning during TAVI.

## Introduction

Transcatheter aortic valve implantation (TAVI) is a relatively novel and highly efficient treatment option for medium- and high-risk patients with aortic stenosis. Short- and long-term survival of patients after TAVI is similar to those after surgical aortic valve replacement (1, 2). The number of TAVI procedures has been steadily growing since the first procedure performed in 2002, and the indications for TAVI continue to expand (3). Minimally invasive procedures are associated with lower mortality and fewer postoperative complications such as atrioventricular block which requires immediate pacing and may cause paraprosthetic leak affecting survival rates (4, 5). Recent studies have reported that specific complications of TAVI are commonly related to a prosthesis-patient mismatch (6–8) and device malpositioning (4). Most peri- and postprocedural complications are operator-dependent but physiological movements of patients during device delivery and deployment may temporarily interrupt the cardiac cycle, limit blood flow, and cause respiratory problems (9, 10). These patient-dependent complications largely depend on the quality of intraoperative imaging which is necessary for accurate device positioning (6). However, routine imaging modalities are limited by the need to reduce the radiologic exposure and to eliminate repeated contrast injections. Therefore, the development of visual assistance systems for intraoperative guidance is of paramount importance.

**Figure 1:**
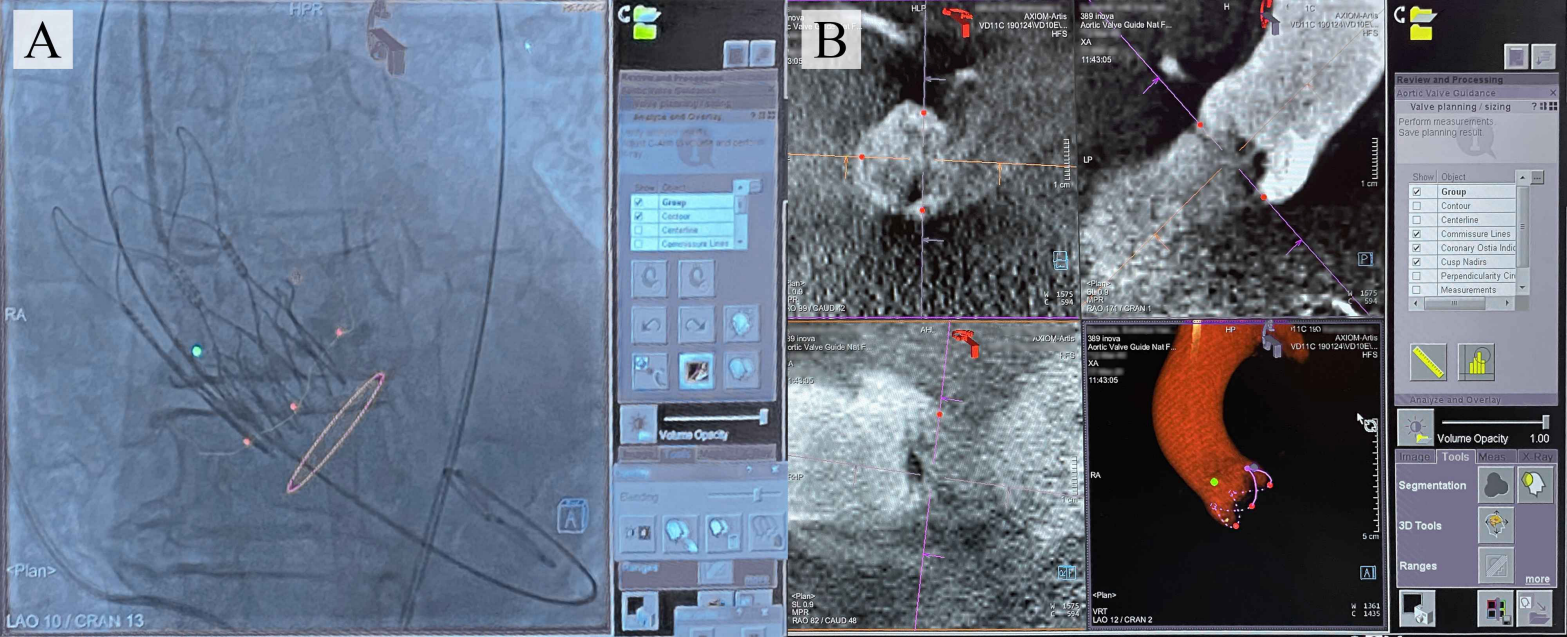
**(A)** Typical images provided by the commercially available TAVI guidance system (Siemens) that delineates the aortic root anatomy, performs its segmentation; **(B)** overlays onto live fluoroscopy, visualizing the key basal hinge points of the leaflets, coronary ostia, the aortic root contour, and suggesting the optimal angiography system orientation.

Several interventional angiography systems integrate commercially available software to facilitate the navigation during TAVI for reducing the risk of complications. To date, such products have been developed by Philips (HeartNavigator), Siemens Healthcare (syngo Aortic Valve Guide), GE Healthcare (Innova HeartVision) (11), and Paieon Inc. (C-THV) (12) and were successfully introduced into clinical practice. The existing guidance systems align the computed tomography (CT)-based 3D anatomical model of the aortic root generated preoperatively and overlay it onto live fluoroscopy images during valve positioning, ensuring the optimal angiography system orientation and vascular access (Figure 1).

However, these systems do not allowreal-time tracking of the keypoints and detailing of the aortic root geometry during TAVI, as they imply preoperative model reconstruction (13). Hence, the operator is still responsible for controlling the position of the device and its deployment by means of the aortography data and pigtail position tracking. The logical step forward is to design visual assistance systems providing an opportunity for the real-time tracking of keypoints and aortic root contour utilizing automated processing of the aortography images, regardless of the image acquisition equipment. For this task, neural networks capable of detecting regions of interest (12, 14) on image series can be employed. Deep learning is currently becoming widespread in cardiovascular imaging (15) for examining aortic root hemodynamics (16, 17), aortic dissection (18), aortic valve biomechanics (19), and coronary artery occlusion (20). Nevertheless, it has not been applied for the valve implantation guidance.

Here, we aimed at developing a tracking system and an algorithm to label the keypoints of the aortic valve anatomical landmarks and TAVI delivery system by using original aortography images obtained during the transcatheter implantation of CoreValve, a self-expanding prosthetic aortic valve, and by applying the multi-task learning (MTL). Previously, MTL has been successfully used in medical imaging (21), computer vision (22, 23), and drug discovery (24). In contrast to single-task learning (STL), MTL acts as a regularizer by introducing an inductive bias, thereby reducing the risk of overfitting as well as the Rademacher complexity of the model, i.e., its ability to fit random noise (25). The ability of the MTL model to find an efficient data representation minimizing the overfitting directly depends on the number of tasks.

## Materials and Methods

The development of the tracking system and labeling algorithm consisted of three main stages:

- Stage 1. Data preparation: data labeling for developing training and validation sets; image annotation by an interventional cardiologist.
- Stage 2. Data analysis: estimation of the distribution of the labels and coordinates of the keypoints.
- Stage 3. Training and screening of neural networks: selection of available neural network architectures, loss function and descriptive metrics, assessment of qualitative and quantitative parameters from the training and validation data.

### Source Data

Original aortography imaging series collected during the implantation of 14 CoreValve self-expanding aortic valve bioprostheses to patients with aortic valve stenosis from 2015 to 2018 were used as the source data for training and validation of neural networks. All TAVI procedures (Table 1) were performed by the same operator at the Department of Cardiovascular Surgery within the Research Institute for Complex Issues of Cardiovascular Diseases.

**Table 1:** Demographic and clinical data of the patients who underwent TAVI procedures.

**Parameter**	**Value**
Total number of procedures	14
Mean age (mean ± SD), years	76.3 ± 5.8
Male patients, *n* (%)	5 (35.7%)
Female patients, *n* (%)	9 (64.3%)
**Prosthesis size**	
26 mm, *n* (%)	6 (42.9%)
29 mm, *n* (%)	7 (50%)
31 mm, *n* (%)	1 (7.1%)
Transfemoral access, *n* (%)	14 (100%)

**Figure 2:**
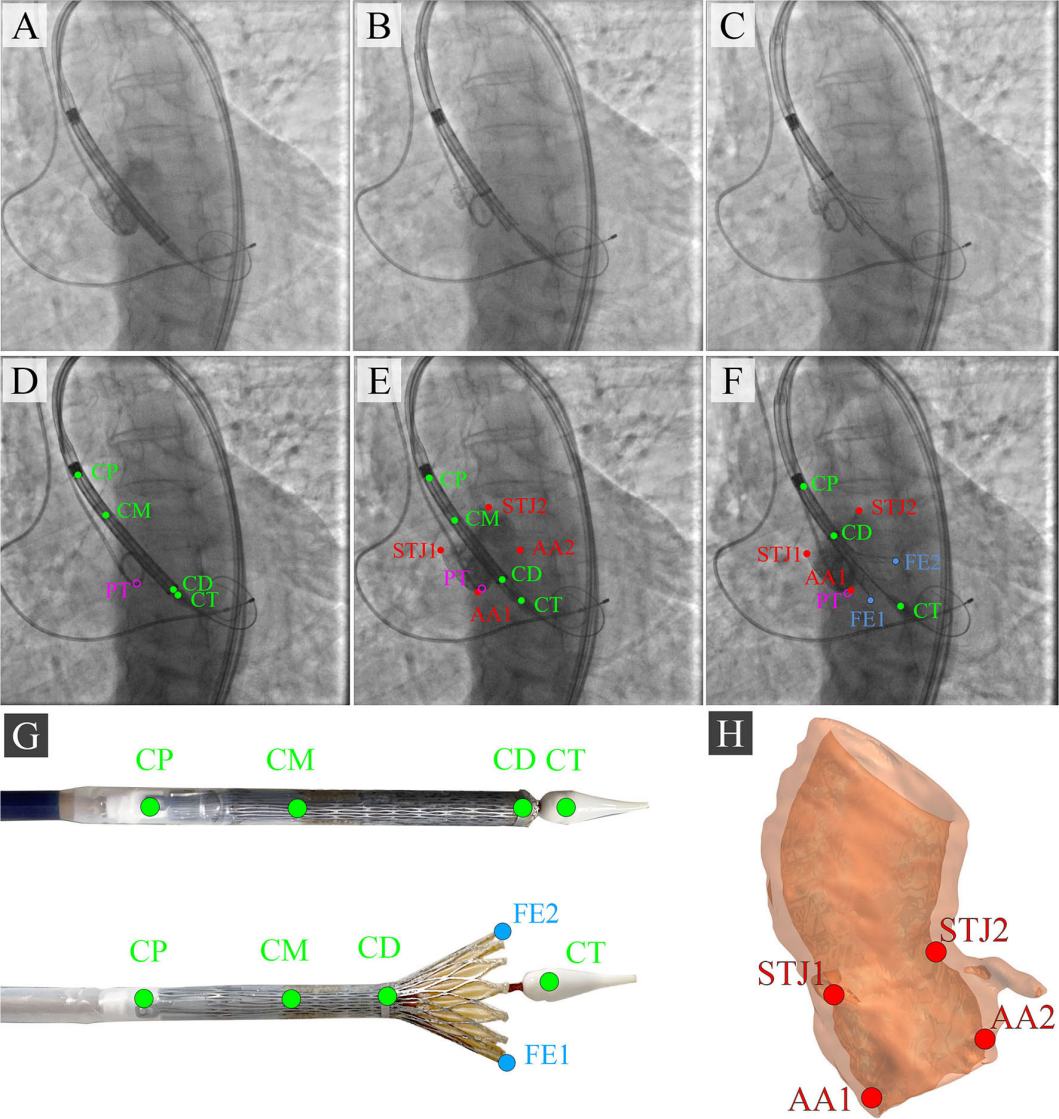
Algorithm for labeling intraoperative aortography images and defining the keypoints for the TAVI tracking system. **(A)** represents the positioning of the delivery system; **(B)** represents the transcatheter aortic valve deployment and the actuator rotation; **(C)** highlights the 1/3 of the valve deployment; **(D)** shows the labeling of the keypoints on the catheter; **(E)** shows the labeling of the keypoints indicative of the aortic root; **(F)** shows the labeling of the keypoints on the valve stent at the stage of its 1/3 deployment; **(G)** is a visualization of the keypoints on the distal part of the delivery system according to the segmented aortograms; **(H)** is a 3D model of the target aortic valve structure.

During the TAVI, we collected 35 video series of 1,000 × 1,000 pixels with an 8-bit depth (a scale from 0 to 255). The final sample consisted of 3,730 grayscale images, of which 2,984 (80%) images were used as the training set and 746 (20%) images were used as the validation set. TAVI allowed obtaining a series of anonymized images illustrating three essential steps: positioning of the catheter and delivery system (Figure 2A); beginning of the capsule retraction and exposing the prosthesis (Figure 2B); deployment of the prosthesis (Figure 2C). The maximum of 11 keypoints of interest (from 1 to 11 over each image) was labeled and annotated (Figures 2D–H). A brief description of the keypoints is provided below.

#### Anatomical Landmarks

- Aortic annulus, a target landmark for TAVI: Aortic root 1 (AA1) and Aortic root 2 (AA2).
- Aortic sinotubular junction, an additional landmark for correct determination of the aortic annulus plane: Sinotubular junction 1 (STJ1) and Sinotubular junction 2 (STJ2).

#### Delivery System Landmarks

- Delivery system anchors, a landmark defining the degree of prosthesis extraction: Catheter Proximal (CP).
- Bending point of the catheter, a landmark of the sinotubular portion of the stent: Catheter Middle (CM).
- The radiopaque capsule marker band on the upper shaft portion to the distal ring, a landmark of the outer shaft bending degree used for defining the extent of prosthesis extraction: Catheter Distal (CD).
- Catheter tip, a landmark determining the location of the catheter and aortic annulus plane: Catheter Tip (CT).

#### Additional Landmarks

- Distal part, a landmark for the valve implantation indicating an aortic annulus plane: Pigtail (PT).
- The distal portion of a self-expanding prosthesis determines the location of the stent during implantation and its deviation from an aortic root plane: Distal part of the stent: Frame Edge 1 (FE1) and Frame Edge 2 (FE2).

To visualize three sequential steps in Figures 2A–C, we selected imaging series during the contrast injection. Data labeling was performed using the Supervisely AI platform.

### Description of the Neural Networks

**Figure 3:**
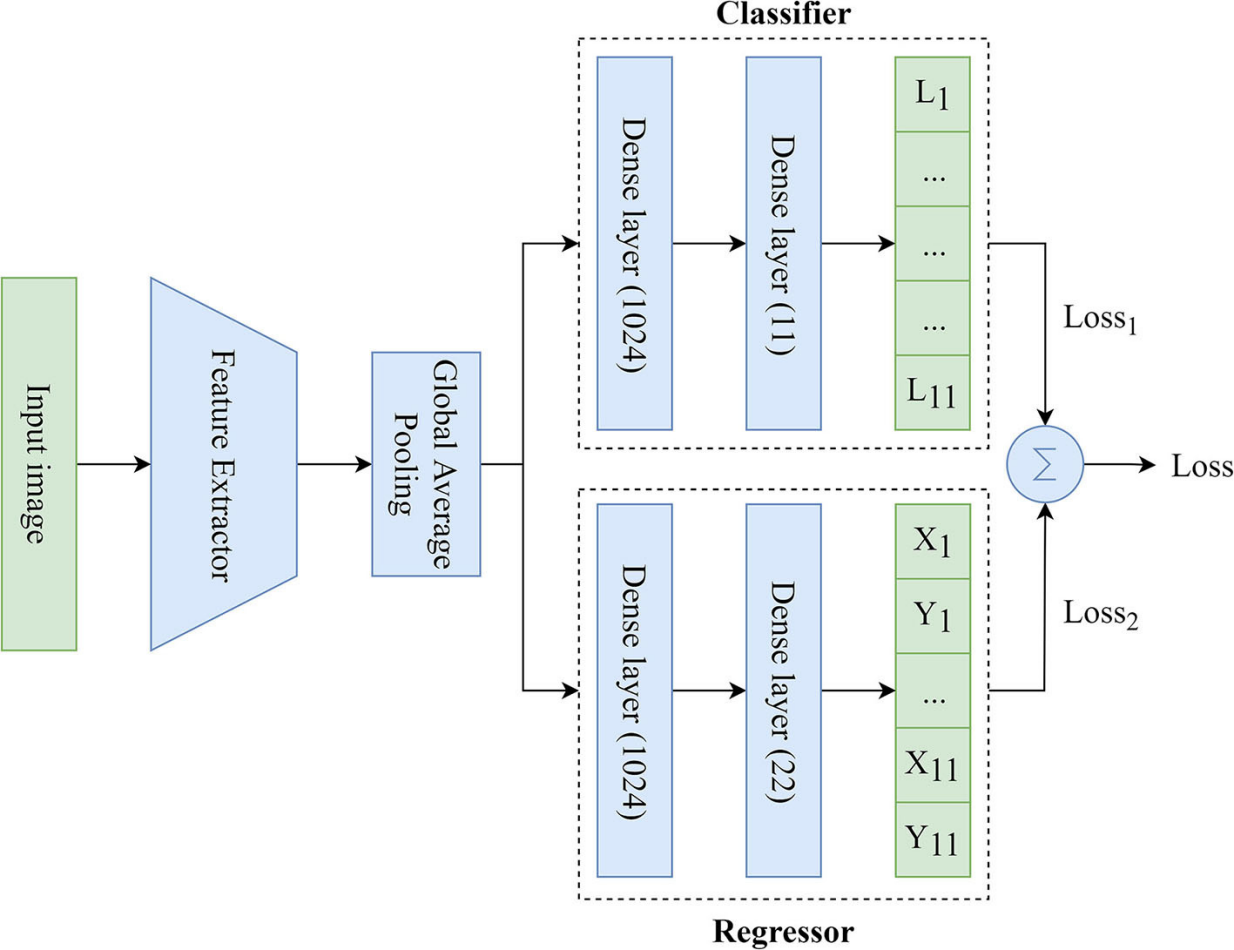
An illustration of the proposed MTL model predicting the labels and coordinates of the keypoints.

We used MTL (26) based on the Hard Parameter Sharing because of the need to simultaneously predict the labels and coordinates of the keypoints. To solve this task, the MTL-based model included three main components (Figure 3):

- Feature Extractor: the component responsible for delineating features and converting them into the lower dimension, i.e., an input image (input tensor) is converted into a vector of features. This vector (output tensor) is a set of optimal descriptors. The dimension of the output tensor is much less than the dimension of the input tensor.
- Classifier: the component responsible for predicting the labels of the keypoints over the image. The output vector of the classifier has 11 outputs, reflecting the probabilities of detecting the keypoints of interest over the image. Since the images contained a different number of points independently of each other, the classifier performed multi-label classification. Thus, the task of the classifier was to determine the keypoints (from 1 to 11) on the image and predict their probabilities. Technically, the multi-label classification task is to find a model that automatically maps an input example to the correct binary vector rather than scalar values.
- Regressor: the component responsible for predicting the coordinates of the keypoints on the image. The output vector of the regressor has 22 outputs, representing the normalized (*x, y*) coordinates of the keypoints of interest on the image.

We applied available neural networks that extract features and implement the abovementioned approach in image processing (Table 2). Training of neural networks was performed with and without fine-tuning. Fine-tuning implied training all parts of the network (feature extractor, regressor, and classifier). Without the fine-tuning, training was performed exclusively for regressor and classifier. Fine-tuning significantly increased the number of weights and the training time.

**Table 2:** Description of the neural networks.

**Model**	**Fine-tuning**	**Size of the input tensor**	**Size of the output tensor**	**Number of trainable parameters**	**Number of non-trainable parameters**	**Total number of parameters**	**Model size, Mb**	**References**
MobileNet V2	Yes	224 × 224 × 3	1280	4,881,185	34,112	4,915,297	58	(27)
	No			2,657,313	2,257,984		40	
ResNet V2	Yes	224 × 224 × 3	2048	27,749,537	45,440	27,794,977	326	(28, 29)
	No			4,230,177	23,564,800		142	
Inception V3	Yes	299 × 299 × 3	2048	25,905,322	34,432	25,939,754	305	(30)
	No			2,085,921	23,853,833		118	
Inception ResNet V2	Yes	299 × 299 × 3	1536	57,900,650	60,544	57,961,194	681	(31)
	No			2,085,921	55,875,273		244	
EfficientNet B5	No	456 × 456 × 3	2048	4,230,177	28,513,520	32,743,697	162	(32)

### Neural Network Training

Since the MTL-based models solve several tasks (e.g., multi-label classification and regression), their training requires the optimization of multiple loss functions. In our study, the generic loss function was the weighted sum of binary cross-entropy (multi-label classification loss function) and Log-Cosh (regression loss function). It was calculated as follows:

(1) $$\begin{array}{r} Loss=w_{1}\cdot Loss_{1}\;+w_{2}\cdot Loss_{2} \end{array}$$

(2) $$Loss_{1}=-\frac{1}{N}\sum_{i=1}^{N}y_{i}\cdot \log{\hat{y}}_{i}+(1-y_{i})\cdot \log(1-{\hat{y}}_{i})$$

(3) $$\begin{array}{r} Loss_{2}=\overset{N}{\underset{i=1}{\sum }}\log\left[\cosh\left({ŷ}_{i}-y_{i}\right)\right]=\overset{N}{\underset{i=1}{\sum }}\log\left(\frac{e^{\left({ŷ}_{i}\;-\;y_{i}\right)}+e^{-\left({ŷ}_{i}\;-\;y_{i}\right)}}{2}\right)\; \end{array}$$

where *y*_*i*_ is the ground-truth value, ŷ_*i*_ is the model prediction, *N* is the number of classes/points. Since the contribution of Log-Cosh to the generic loss function is much less, the value of the weight *w*_2_ was chosen equal to 10, and the value of the weight *w*_1_ was chosen equal to 1 to maintain the balance.

We have chosen Log-Cosh because it combines the advantages of both Mean Absolute Error (MAE) and Mean Squared Error (MSE) loss functions. This loss function is approximately equal to |ŷ_*i*_ − *y*_*i*_| − log(2) for large values of the prediction error and ${\left({ŷ}_{i}\;-\;y_{i}\right)}^{2}\;/\;$2 for small values of the prediction error. Unlike MSE, Log-Cosh is less sensitive to random incorrect predictions or outliers. It also has all the advantages of Huber loss. Importantly, Log-Cosh is twice differentiable and may be used in several specific machine learning models [e.g., many ML solutions like XGBoost use Newton's method to find the optimum, where the second derivative (Hessian) is needed].

Early Stopping, a form of regularization, was used to avoid the model overfitting. The training of the model was terminated once the model performance stopped improving at least 0.005 during 5 epochs on a hold-out validation set. To train the models, we used the Rectified Adam (33) with a learning rate of 0.00001 and a batch size of 64.

All neural networks were trained using Intel Core i7-4820K 3.7 GHz CPU, 32 Gb RAM, NVIDIA GeForce RTX 2080 Ti 11 Gb, Ubuntu 18.04.4 LTS (Bionic Beaver). We selected the following metrics to assess classification and regression components of the neural networks:

#### Classification Metrics

(4) $$\begin{array}{r} Precision=\frac{TP}{TP+FP} \end{array}$$

(5) $$\begin{array}{r} Recall=\frac{TP}{TP+FN} \end{array}$$

(6) $$\begin{array}{r} F_{1}=\frac{2\cdot TP}{2\cdot TP+FN+FP} \end{array}$$

(7) $$\begin{array}{r} Accuracy=\frac{TP+TN}{TP+FN+TN+FP} \end{array}$$

#### Regression Metrics

(8) $$\begin{array}{r} MAE=\frac{1}{N}\;\overset{N}{\underset{i=1}{\sum }}\;|y_{i}\;-{\;ŷ}_{i}| \end{array}$$

(9) $$\begin{array}{r} MSE=\frac{1}{N}\;\overset{N}{\underset{i=1}{\sum }}\;{\left(y_{i}\;-{\;ŷ}_{i}\right)}^{2} \end{array}$$

(10) $$\begin{array}{r} RMSE=\sqrt{\frac{1}{N}\;\overset{N}{\underset{i=1}{\sum }}\;{\left(y_{i}\;-{\;ŷ}_{i}\right)}^{2}} \end{array}$$

where *TP* is the number of true positives, *TN* is the number of true negatives, *FP* is the number of false positives, *FN* is the number of false negatives, *y*_*i*_ is the ground-truth value, ŷ_*i*_ is the predicted value, *N* is the number of samples.

We use the general method for computing the *F*1*-*score (Eq. 6). The micro-*F*1 represented the total number of *TP, FN*, and *FP*. The macro-*F*1 was a weighted average of the *F*1 scores of each class.

### Software Used in the Study

During the performance of our study, we used several key libraries, packages, and frameworks such as:

- Python Version 3.6.9 (RRID:SCR_008394) and PyCharm Version 2020.1 (RRID:SCR_018221) were used as the main programming language and integrated development environment for performing data processing/wrangling and neural networks development;
- R Version 3.6.3 (RRID:SCR_001905) and RStudio Version 1.2.5001 (RRID:SCR_000432) were used as an additional programming language and integrated development environment for performing statistical analysis;
- TensorFlow (RRID:SCR_016345) is an open-source software library used for the development of the deep learning networks trained using the MTL approach;
- Scikit-learn Version 0.20.3 (RRID:SCR_002577) is an open-source software machine learning library for the Python programming language;
- SciPy Version 1.4.1 (RRID:SCR_008058) is an open-source library for the scientific computing including numerical integration, interpolation, optimization, linear algebra, and statistics;
- NumPy Version 1.18.2 (RRID:SCR_008633) is a numerical computing tool used for processing multi-dimensional arrays and matrices;
- Pandas Version 0.24.2 (RRID:SCR_018214) is a software library for data manipulation and analysis of different data structures including numerical tables and time series;
- OpenCV Version 4.0.1.23 (RRID:SCR_018214) is a computer vision library used for image processing and visualization;
- Seaborn Version 0.10.0 (RRID:SCR_018132) and Matplotlib 3.0.3 (RRID:SCR_008624) are comprehensive libraries for creating static, animated, and interactive visualizations in Python;
- ggplot2 Version 3.2.1 (RRID:SCR_014601) is a data visualization package for the statistical programming language R.

## Results

### Analysis of the Source Data

**Figure 4:**
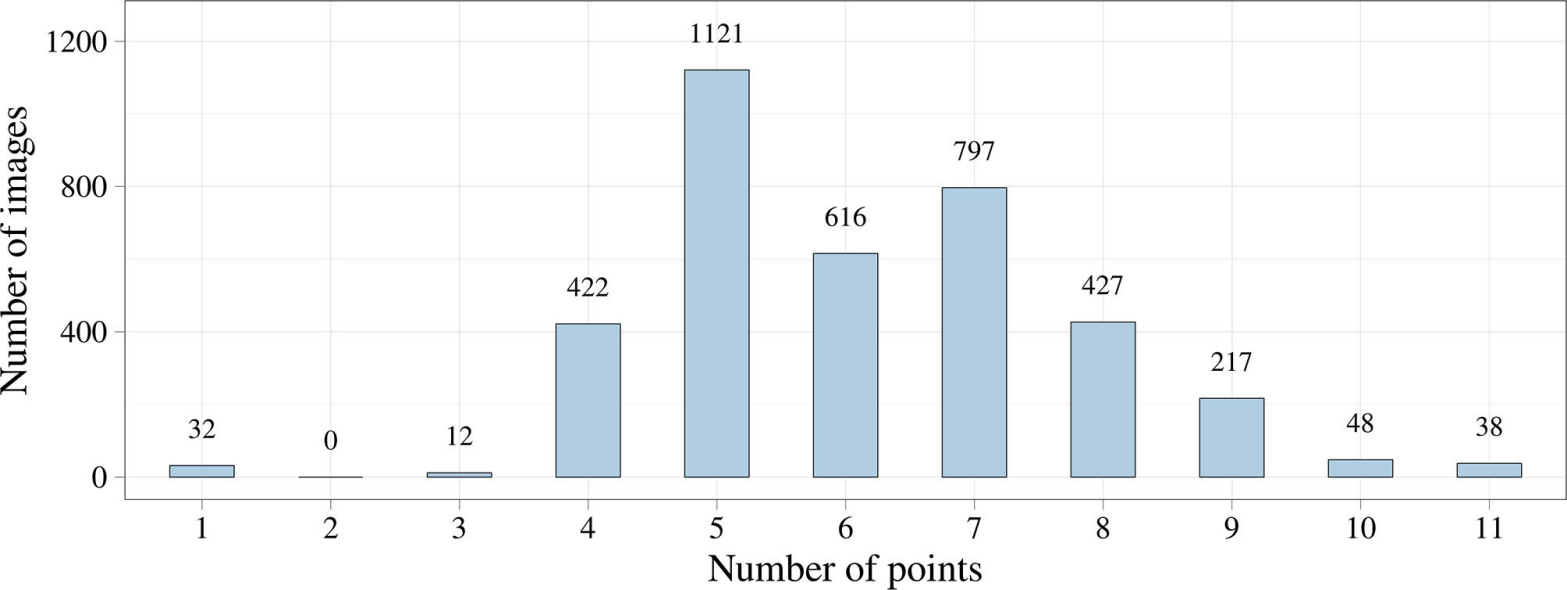
Distribution of the number of keypoints in the initial dataset.

We first analyzed the distribution of the keypoint number using exploratory data analysis. Figure 4 shows that the number of keypoints on images has a normal distribution. However, we noticed the imbalance of the initial dataset due to a small number of images where the keypoints of the aortic valve landmarks and TAVI delivery system were visualized during contrasting. This imbalance could affect the predictive power of the models but may be eliminated by increasing the number of images of the minor class. In some cases, images containing 1, 2, 3, 10, and 11 keypoints of interest can be predicted incorrectly.

**Figure 5:**
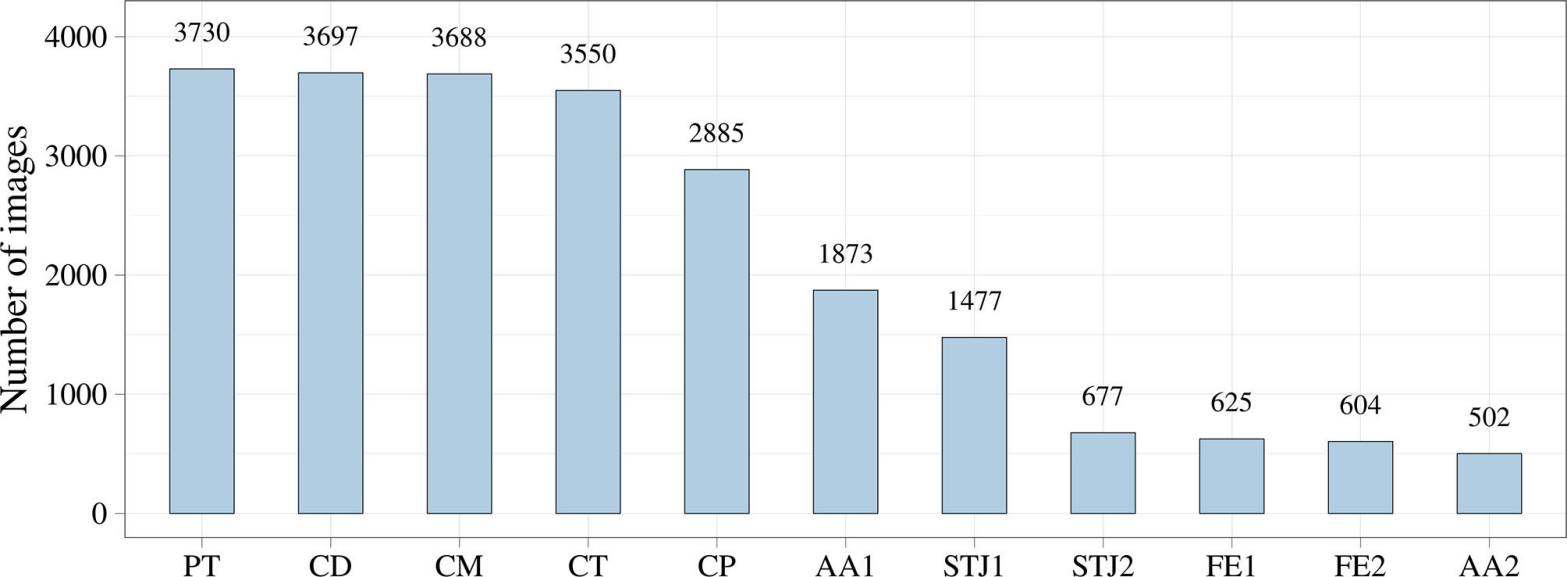
The total number of images for the studied keypoints.

**Figure 6:**
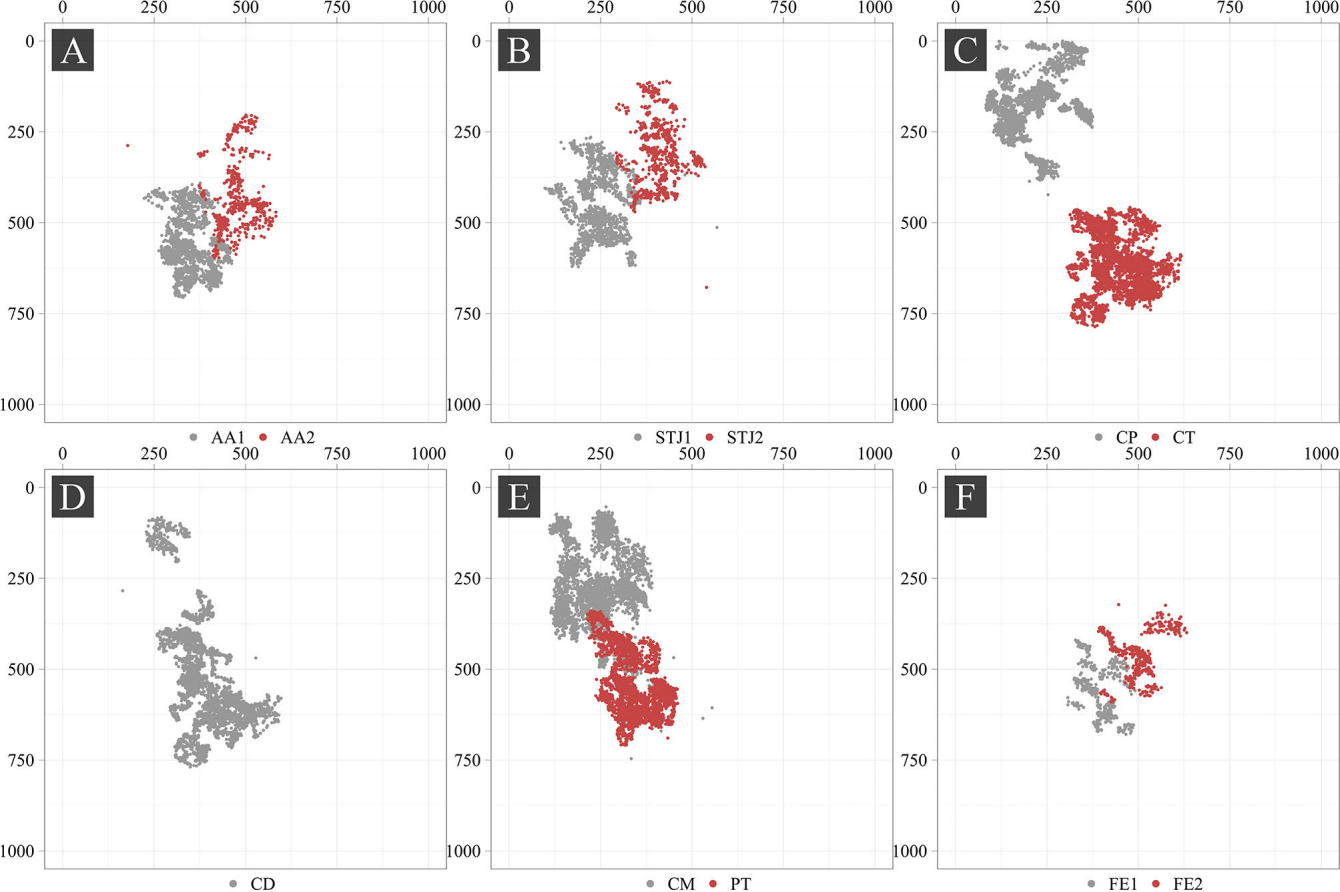
Scatter plots of the keypoints in the source images.

Additionally, we analyzed the distribution of the keypoints in the images (Figure 5). Most of the keypoints represented the delivery system (CP, CM, CD, and CT) and pigtail (PT). There were fewer keypoints of anatomical landmarks (AA2 and STJ2) and distal portion of the stent (FE1 and FE2) that can be explained by a limited imaging time during the TAVI procedures. Most of the analyzed images were made without contrasting that prohibited the visualization of the keypoints indicating aortic valve anatomical landmarks (AA1, AA2, STJ1, and STJ2). Since the valve is pre-attached to the delivery system, FE1 and FE2 were tracked only at the last stage of the procedure. Thus, the classifier may be biased toward predicting the majority class (PT, CD, CM, CT, and CP). To assess the distribution of the keypoint coordinates, scatter plots were used (Figure 6).

Point cloud density and data scatter of the distal portion of the stent (FE1 and FE2) displayed a small number of these points, suggesting the presence of the imbalance in the source dataset. We noted the presence of the statistical outliers, i.e. single points that are shown in Figure 6 (AA2, STJ1, STJ2, CD, CM, and CP). In addition, the keypoints of interest were distributed unevenly over the images and localized in small areas. The latter, theoretically, can lead to the memorization of the coordinates by the model, resulting in poor accuracy on the validation set. In case the model is overfitting, the augmentation of images using affine or geometric transformations (image rotation, reflection, translation, etc.) may be applied.

### Neural Network Training

**Figure 7:**
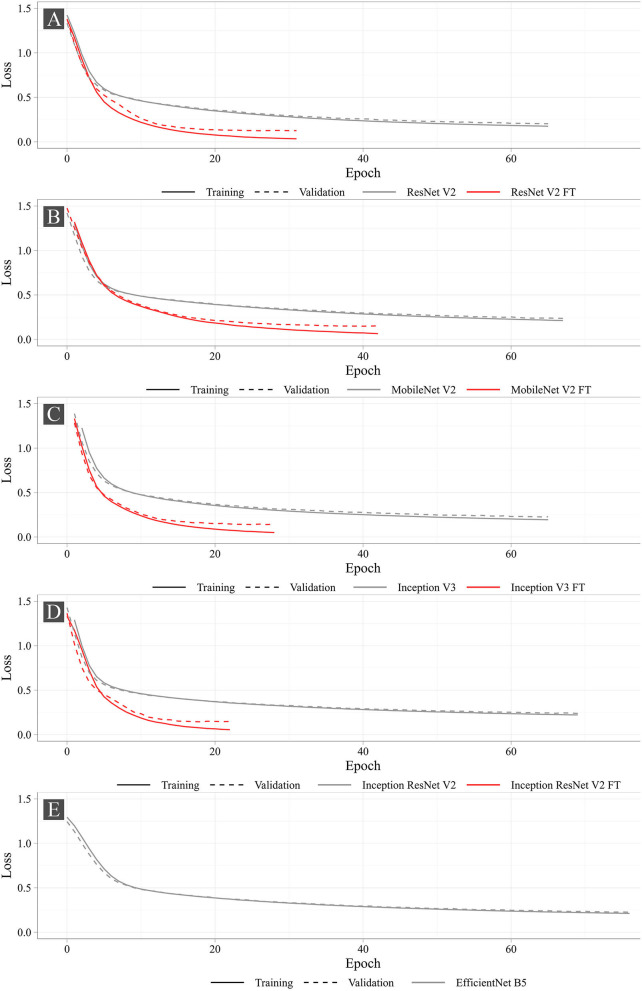
Dynamic changes in the loss function for the studied neural networks.

Figure 7 shows the training progress of the studied neural networks. The graphs present the dynamic changes in the values of the loss function for both fine-tuned and non-fine-tuned models. The dashed line shows the dynamic changes in the loss function on the validation set. Despite the number of epochs for training was set to 100, none of the models reached the set number. The largest number of epochs spent in training was 76 (EfficientNet B5), the smallest was 22 (Inception ResNet V2 FT). According to the loss function analysis, it was shown that fine-tuned models were more prone to overfitting (Supplementary Table 1) that is typical for all fine-tuned models. However, Early Stopping allowed partial elimination of the model overfitting. Importantly, heavier models (Inception ResNet V2 and EfficientNet B5) were less likely to overfit.

**Figure 8:**
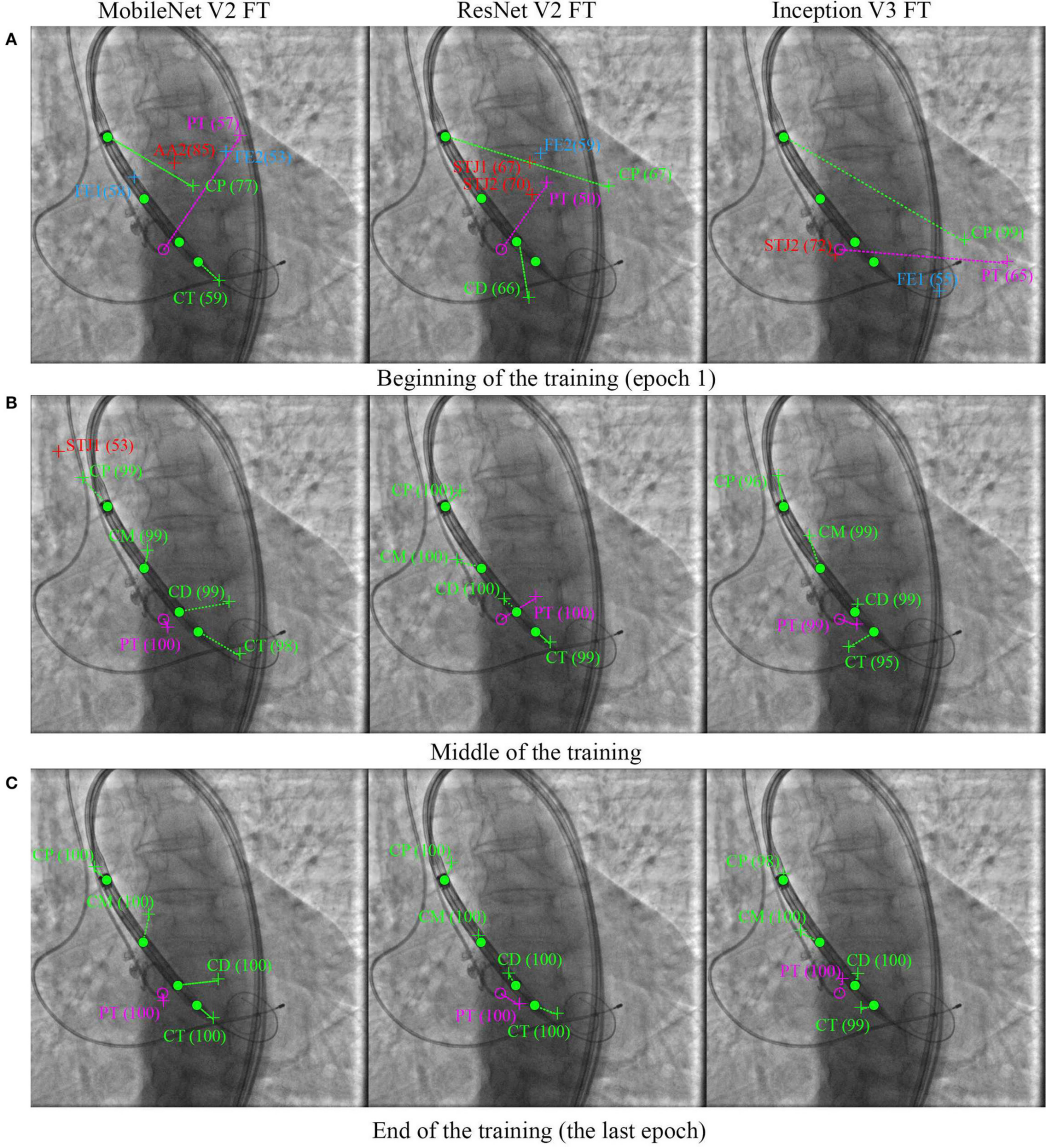
Prediction of the keypoint labels and coordinates. Dots are the ground-truth keypoints defined by the expert. Crosses are the predictions of neural networks. Ideally, the number and the coordinates of the ground-truth and predicted keypoints should coincide. **(A)** Beginning of the training (epoch 1), **(B)** middle of the training, and **(C)** end of the training (the last epoch).

Figure 8 shows the learning dynamics of three models, MobileNet V2 FT, ResNet V2 FT, and Inception V3 FT. After the initial weights were initialized and the models were trained for one epoch, they predicted the labels and the keypoint coordinates incorrectly. By the middle of the training, almost all models performed classification and regression with a high degree of accuracy, except some models did not reach their optimum in training [e.g., MobileNet V2 FT still predicted the presence of STJ1 in the image with a probability of 53% (Figure 8B)]. By the end of the training, all models predicted the presence of keypoints and their coordinates over the images with a fairly low error rate (Figure 8C).

### Quantitative Analysis of the Models

After the training process, we compared the selected metrics described in the Materials and Methods section. Tables 3, 4 show the results of the comparative analysis. Color scale formatting reflects the distribution of models by their accuracy, where deep blue shows a better prediction, and white indicates a worse prediction. All metrics are normalized in the range [0; 1].

**Table 3:** Model comparison according to the classification metrics^*^.

**Model**	**Precision**		**Recall**		**Macro** ***F*****1**		**Micro** ***F*****1**		**Accuracy**	
	**Train**	**Valid**	**Train**	**Valid**	**Train**	**Valid**	**Train**	**Valid**	**Train**	**Valid**
ResNet V2 FT	0.99	0.97	1.00	0.97	0.99	0.93	1.00	0.97	0.99	0.97
ResNet V2	0.97	0.96	0.95	0.95	0.89	0.89	0.96	0.95	0.96	0.95
MobileNet V2 FT	0.99	0.96	0.99	0.97	0.98	0.92	0.99	0.96	0.99	0.96
MobileNet V2	0.96	0.95	0.94	0.93	0.86	0.84	0.95	0.94	0.95	0.93
Inception V3 FT	0.99	0.96	1.00	0.97	0.99	0.93	0.99	0.97	0.99	0.96
Inception V3	0.96	0.95	0.94	0.93	0.88	0.85	0.95	0.94	0.95	0.94
Inception ResNet V2 FT	0.99	0.96	0.99	0.97	0.98	0.92	0.99	0.97	0.99	0.96
Inception ResNet V2	0.96	0.95	0.93	0.92	0.85	0.82	0.94	0.94	0.94	0.93
EfficientNet B5	0.96	0.96	0.93	0.93	0.86	0.86	0.95	0.94	0.94	0.94

* *The higher the metric value, the better the model predicts the labels of the keypoints*.

**Table 4:** Model comparison according to the regression metrics^*^.

**Model**	**MAE**		**RMSE**		**MSE**	
	**Train**	**Valid**	**Train**	**Valid**	**Train**	**Valid**
ResNet V2 FT	0.035	0.047	0.049	0.079	0.002	0.006
ResNet V2	0.062	0.067	0.092	0.100	0.009	0.010
MobileNet V2 FT	0.045	0.056	0.066	0.088	0.004	0.008
MobileNet V2	0.070	0.074	0.102	0.109	0.010	0.012
Inception V3 FT	0.049	0.053	0.070	0.086	0.005	0.007
Inception V3	0.068	0.074	0.100	0.108	0.010	0.012
Inception ResNet V2 FT	0.048	0.046	0.070	0.082	0.005	0.007
Inception ResNet V2	0.071	0.073	0.105	0.109	0.011	0.012
EfficientNet B5	0.065	0.067	0.100	0.103	0.010	0.011

* *The lower the metric value, the better the model predicts the coordinates of the keypoints*.

**Figure 9:**
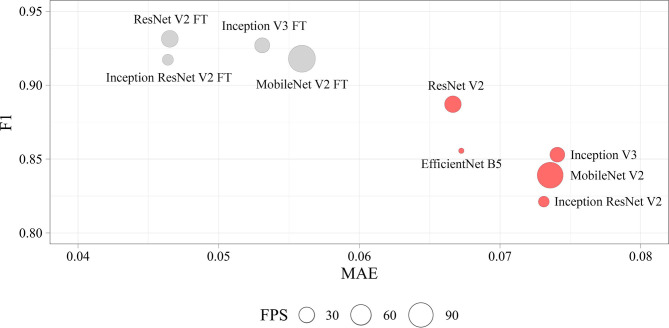
Visualization of the key performance indicators of neural networks. Each circle represents the performance of the model in terms of frames per second on the validation set (the larger the circle, the higher the model prediction speed).

We determined four models (ResNet V2 FT, MobileNet V2 FT, Inception V3 FT, and Inception ResNet V2 FT) that were capable of performing both multi-label classification and regression with high accuracy. Fine-tuning better solved the set tasks by demonstrating the best performance, *F*1-score, and MAE (Figure 9). These models demonstrated a higher generalization capability than standard models, better extending the dependencies and patterns found on the training set to the validation set. However, fine-tuned models are more prone to overfitting and may require the introduction of additional regularizers.

### Time Analysis of the Models

To assess the efficiency of the selected approach, we assessed the training time and the prediction time of each model. We found a strong positive correlation between the number of weights and the training/prediction time. Importantly, fine-tuned models trained twofold longer than non-fine-tuned ones. However, fine-tuned models converged faster, leading to fewer epochs for training. A detailed comparison of the time metrics in relation to the selected models is shown in Supplementary Table 2.

## Discussion

Our approach to the tracking of the intraoperative data using a unique labeling algorithm represents a novel software that may improve clinical outcomes of patients undergoing TAVI. To better evaluate the reliability of the results, we should distinguish two primary indicators: precise, real-time operation of the algorithm and its high accuracy. Theoretically, the performance of this software can be compared with the previously described TAVI imaging software solutions (HeartNavigator, syngo Aortic Valve Guide, etc.). However, this comparison cannot be conducted in real clinical settings since all commercially available imaging software solutions are used for the preoperative planning and vascular access rather than for the intraoperative guidance as an additional imaging modality. Our tracking software facilitates the valve implantation, guiding the operator to adequate valve positioning and deployment. Therefore, it is reasonable to discuss specific parameters that may prove its efficiency and safety. For instance, frame per second indicator is critical for neural network software solutions but not for routine imaging modalities. Future research may focus on the validation of the intraoperative modalities for tracking aortic valve anatomical landmarks using clinical or mixed data.

In comparison with a hard parameter sharing utilized in our study, an ensemble of soft parameter sharing MTL-generated models may reduce coordinate scattering and increase the generalization capability of the approach. However, surgical interventions require real-time data processing, limiting the pool of the models that can be applied. In addition, the use of time-distributed architecture for our neural network ensemble permitted involvement of both spatial and temporal components to reduce oscillations of the keypoint coordinates.

**Figure 10:**
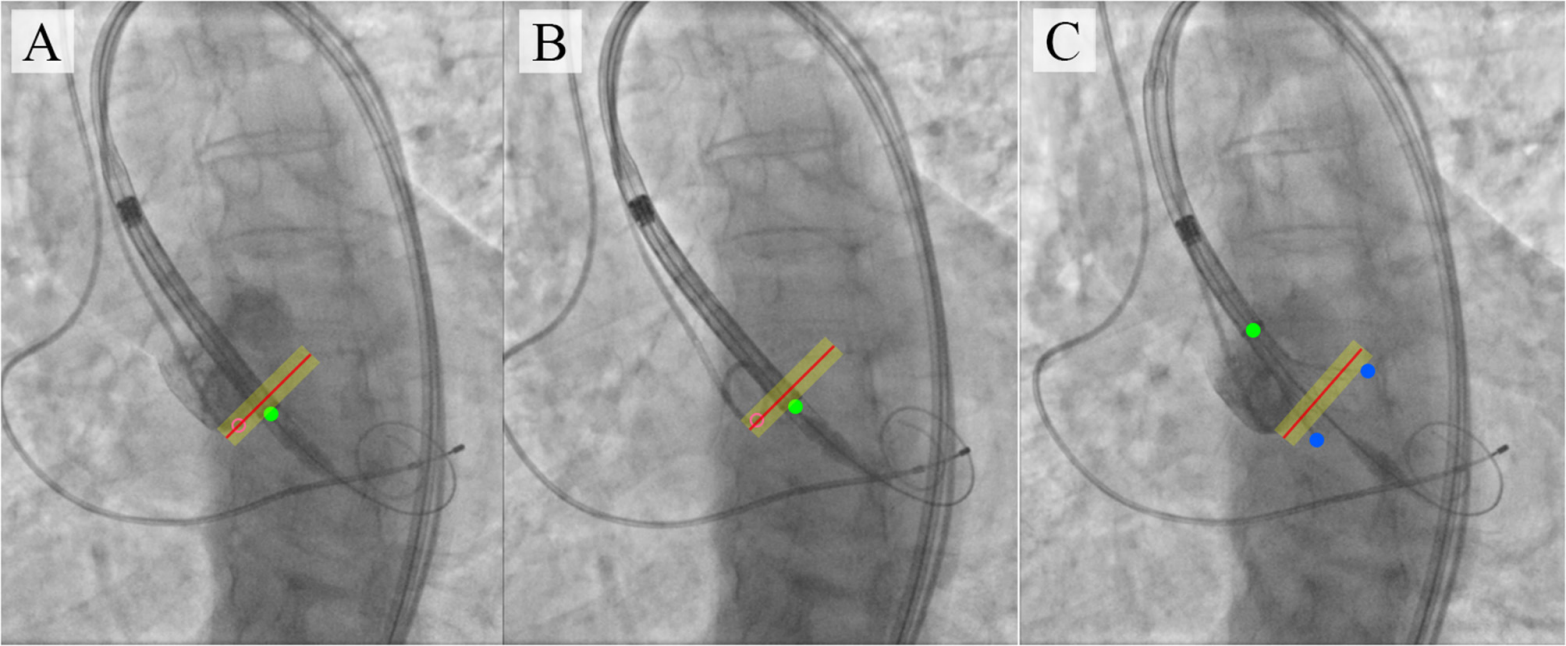
An illustration of TAVI visual assistance system output generated by the proposed algorithm. **(A–C)** visualize the catheter location and the target implantation site, where **(A)** is a target implantation site tracked by the pigtail location during the contrast injection; **(B)** is a tracking of catheter location relative to the aortic annulus plane with an acceptable implantation error in the absence of contrast; **(C)** is an example of imaging with partial extraction of the valve from the delivery system.

The proposed algorithm and its further optimization will allow to develop a virtual TAVI assistant capable of providing relevant information to interventional cardiologists (Figure 10). Tracking and labeling of 11 keypoints within the aortic root and TAVI delivery system will support the operator in determining the intraoperative deviation of the delivery system from the optimal trajectory recommended by the manufacturer. Further, it will perform real-time visualization of the target implantation site and TAVI delivery system based on the algorithmic binding of the pigtail catheter to the coordinates without the need for repeated contrasting (Figure 10C).

Another promising research area is the development of a feedback loop for robotic-assisted TAVI systems that have been designed for experimental purposes (10, 34). The main concept of this approach is the use of manipulators compatible with the commercial TAVI systems that would deliver and position valves instead of interventional cardiologists, who will then monitor and control the work of the robotic assistant. The performance of these systems depends on the input parameters from the angiography system to control real-time tracking of the catheter location and aortic valve anatomical landmarks. In this respect, our neural network ensemble for the real-time tracking of 11 keypoints is a source of the input data for the hardware complexes of the robotic assistants that perform semi-automated TAVI procedures.

**Figure 11:**
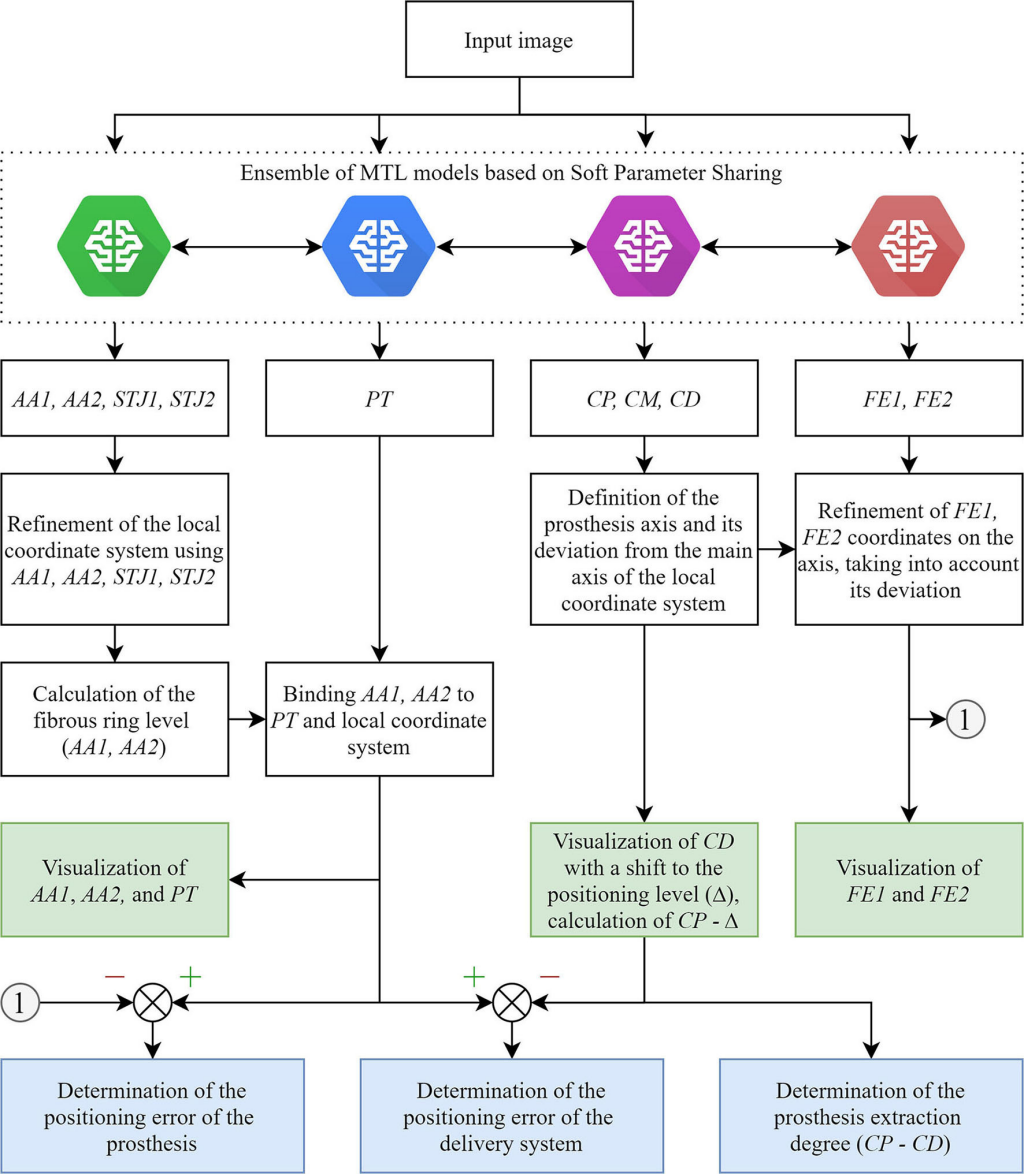
An illustration of an updated algorithm for tracking and labeling the keypoints of the aortic valve and TAVI delivery system.

The main limitation of the real-time tracking in this study was the relatively high error in predicting the keypoint coordinates due to a small number of images with aortic valve anatomical landmarks (AA2 and STJ2) and the distal portion of the stent (FE1 and FE2). The pixel distance between predicted and ground-truth points varied from 40 to 60 pixels with an image size of 1,000 × 1,000 pixels. Therefore, our further studies will be focused on optimizing the MTL-based algorithm for imbalanced datasets (Figure 11) that will guide the operator for optimal valve positioning. The algorithm is based on the tracking of 11 keypoints: the aortic root (AA1, AA2, STJ1, STJ2), pigtail (PT), delivery system (CP, CM, CD), and transcatheter valve (FE1, FE2). Tracking the aortic root during contrasting, the algorithm generates a local orthogonal coordinate system in two dimensions, where AA1 and AA2 keypoints form the *X*-axis (aortic annulus plane) perpendicular to the *Y*-axis. Once the contrast injection has passed and these points cannot be longer tracked, PT acts as a duplicating element suggesting the origin of coordinates and ensuring the binding of AA1 and AA2 to PT. Simultaneously, the algorithm tracks and labels the keypoints of the catheter (CP, CM, CD), providing relevant information to the TAVI operator for the proper positioning of the delivery system and starting valve deployment. FE1 and FE2 indicated the outer shaft of the delivery system, suggesting the accuracy of valve positioning and any potential dislodging from the aortic annulus plane. Thus, our software performs a two-stage assessment of the errors that may occur during valve positioning and deployment (i.e., “annulus-catheter” and “annulus-stent” coordinate difference). In addition, CP-CD keypoints provide relevant information on the extraction degree of the outer shaft.

Despite relatively small sample size might limit the quality of neural network training, the selected neural network architectures and learning approach resulted in <5% mean absolute error for both classification and regression functions in training and validation samples. The single-center single-operator design is another limitation of this investigation. Yet, we think that it is acceptable in the proof-of-concept study which suggests a novel experimental tool rather than an instrument for the direct implementation into cardiovascular surgery. Despite an extensive search, we could not find any studies regarding the application of any convolutional neural network algorithm for the real-time tracking of aortic valve and delivery system keypoints during TAVI, even for one patient. Further, in combination with a single-prosthesis (CoreValve, Medtronic) study design a single-operator approach minimizes the sample heterogeneity that is of crucial importance when designing artificial intelligence tools. Implantation of all prosthetic valves by a single operator excluded variability of the technique and increased the precision of machine learning, thereby contributing to the accuracy of the algorithm. Among all commercially available prosthetic valves, we selected CoreValve with regards to: (1) a large amount of research regarding this valve prosthesis model, including those investigating the correlation between its inadequate positioning and postoperative complications; (2) it has a self-expanding frame similar to most of prosthetic valves employed in TAVI; (3) a specific experience of cardiovascular surgeons in our center. Notwithstanding, we suggest that further investigations should include several models of prosthetic heart valves. In addition, the neural networks designed in this study require validation in a two- or multi-center (and therefore multi-operator) study.

## Conclusion

To summarize, we suggest a novel real-time tracking system for the facilitation of TAVI procedures. Here, we provided a proof of concept that such a system can recognize and track the keypoints indicating the location of the aortic root, delivery system, and heart valve prosthesis during TAVI. Based on the hard parameter sharing, MTL approach ensured the simultaneous, real-time prediction of the keypoint labels and coordinates with an overall accuracy above 95%: fully trained ResNet V2 and MobileNet V2 networks predicted labels with an *F*1-score of 97 and 96%, and coordinates with a mean absolute error of 4.6 and 5.6%, respectively. We suggest these neural networks might be employed both as a supporting tool to optimize valve positioning and as a component of a robotic-assisted system for performing TAVI.

## Data Availability Statement

The dataset presented in this study can be found in the repository of the Research Laboratory for Processing and Analysis of Big Data (Tomsk Polytechnic University): https://www.dropbox.com/sh/80wpfkdabhuo0l9/AADuysNg3sO00_vjhW8MgZ6Ba?dl=0.

## Ethics Statement

The studies involving human participants were reviewed and approved by the Local Ethics Committee of the Research Institute for Complex Issues of Cardiovascular Diseases (approval letter No. 11 issued on June 28, 2018). The patients/participants provided their written informed consent to participate in this study.

## Author Contributions

EO conceived the idea of the study. EO, VD, and KK developed the plan of the study design, wrote the manuscript with input from all the co-authors, and analyzed the performance of deep learning networks on the collected data. KK, EO, VG, and AS acquired the data. VD, IS, and KK prepared the software and algorithms for data analysis. VD developed, trained, and tested deep learning networks. IS, OG, and AK contributed to the methodology. VG and EO were supervising and administering the project. KK and EO contributed critical discussions and revisions of the manuscript. All authors contributed to the article and approved the submitted version.

## Conflict of Interest

The authors declare that the research was conducted in the absence of any commercial or financial relationships that could be construed as a potential conflict of interest.
