# Supplementary material for: Aortography Keypoint Tracking for Transcatheter Aortic Valve Implantation Based on Multi-Task Learning
Source: Front Cardiovasc Med. 2021 Jul 19;8:697737. doi: 10.3389/fcvm.2021.697737 (PMC8326378; doi:10.3389/fcvm.2021.697737)
Supplement: Supplementary file 1 [file Table_1.DOCX]

**Table 1. Comparison of the loss function values for the studied neural network.**

| **Model** | **Epoch number** | **Loss**  **(training)** | **Loss (validation)** | **∆Loss** | |
| --- | --- | --- | --- | --- | --- |
|  |  |  |  | **Abs.** | **Rel.** |
| ResNet V2 FT | 31 | 0.033 | 0.125 | 0.0915 | 275% |
| ResNet V2 | 65 | 0.174 | 0.203 | 0.0283 | 16% |
| MobileNet V2 FT | 42 | 0.065 | 0.153 | 0.0880 | 136% |
| MobileNet V2 | 67 | 0.212 | 0.237 | 0.0245 | 12% |
| Inception V3 FT | 28 | 0.050 | 0.144 | 0.0934 | 185% |
| Inception V3 | 65 | 0.194 | 0.227 | 0.0326 | 17% |
| Inception ResNet V2 FT | 22 | 0.056 | 0.148 | 0.0919 | 164% |
| Inception ResNet V2 | 69 | 0.222 | 0.239 | 0.0170 | 8% |
| EfficientNet B5 | 76 | 0.212 | 0.226 | 0.0136 | 6% |
