# Supplementary material for: Aortography Keypoint Tracking for Transcatheter Aortic Valve Implantation Based on Multi-Task Learning
Source: Front Cardiovasc Med. 2021 Jul 19;8:697737. doi: 10.3389/fcvm.2021.697737 (PMC8326378; doi:10.3389/fcvm.2021.697737)
Supplement: Supplementary file 2 [file Table_2.DOCX]

**Table 2. Comparison of neural networks according to the training and prediction time.**

| **Model** | **Image size** | **Duration of 1 epoch** | **Number of epochs** | **Training time** | **Prediction time, ms** | **FPS** |
| --- | --- | --- | --- | --- | --- | --- |
| ResNet V2 FT | 224x224x3 | 9m 33s | 31 | 4h 56m 32s | 29 | 34 |
| ResNet V2 | 224x224x3 | 2m 07s | 65 | 2h 18m 20s | 30 | 34 |
| MobileNet V2 FT | 224x224x3 | 3m 45s | 42 | 2h 37m 16s | 9 | 115 |
| MobileNet V2 | 224x224x3 | 45s | 67 | 49m 49s | 10 | 105 |
| Inception V3 FT | 299x299x3 | 13m 39s | 28 | 6h 22m 13s | 40 | 25 |
| Inception V3 | 299x299x3 | 2m 50s | 65 | 3h 5m 10s | 40 | 25 |
| Inception ResNet V2 FT | 299x299x3 | 28m 30s | 22 | 10h 27m 13s | 97 | 10 |
| Inception ResNet V2 | 299x299x3 | 6m 46s | 69 | 7h 47m 25s | 97 | 10 |
| EfficientNet B5 | 456x456x3 | 21m 38s | 76 | 27h 25m 30s | 331 | 3 |

* The higher the FPS (frames per second) value, the faster the model make predictions
